# Mental disorders and excess mortality: a systematic review protocol

**DOI:** 10.1136/bmjopen-2024-084797

**Published:** 2025-03-15

**Authors:** Shae E. Quirk, Heli Koivumaa-Honkanen, Amanda L Stuart, Mohammadreza Mohebbi, Risto J Honkanen, Jeremi Heikkinen, Michael Berk, Julie A Pasco, Lana J Williams

**Affiliations:** 1 Deakin University, Institute for Mental and Physical Health and Clinical Translation, School of Medicine, Geelong, Victoria, Australia; 2 Institute of Clinical Medicine, Kuopio Musculoskeletal Research Unit (KMRU), University of Eastern Finland, Kuopio, Finland; 3 Institute of Clinical Medicine, Psychiatry, University of Eastern Finland, Kuopio, Finland; 4 Biostatistics Unit, Faculty of Health, Deakin University, Melbourne, Victoria, Australia; 5 University Hospital Geelong, Barwon Health, Geelong, Victoria, Australia; 6 Department of Psychiatry, Orygen the National Centre of Excellence in Youth Mental Health, Centre for Youth Mental Health, Florey Institute of Neuroscience and Mental Health, The University of Melbourne, Parkville, Victoria, Australia; 7 Department of Medicine – Western Health, The University of Melbourne, St Albans, Victoria, Australia; 8 Department of Epidemiology and Preventive Medicine, Monash University, Melbourne, Victoria, Australia

**Keywords:** Mortality, MENTAL HEALTH, PSYCHIATRY, EPIDEMIOLOGY, Systematic Review

## Abstract

**Abstract:**

**Background:**

There is developing evidence of excess mortality among people with mental disorders. This protocol presents the methodology to undertake a systematic review to definitively examine the current evidence on the risk of all-cause and cause-specific mortality in people with mental disorders (mood, anxiety, substance use, eating, personality and psychotic disorders) compared with populations without mental disorders in broadly representative studies of general populations worldwide. In addition, we seek to understand whether the excess mortality has increased further over time, and if the COVID-19 pandemic exacerbated the excess mortality in people with mental disorders.

**Methods:**

A systematic review of cohort studies will be conducted. The search strategy to yield peer-reviewed (in Medline Complete, CINAHL Complete, Embase and APA PsycInfo) and published grey literature will be developed in consultation with a liaison librarian. A preliminary scope of peer-reviewed literature in Medline Complete using the EBSCOhost platform was conducted on 20 November 2023. Epidemiological cohort or case-control studies will be eligible if they examine (1) diagnoses of mental disorders (according to the Diagnostic and Statistical Manual of Mental Disorders and the International Classification of Diseases classification systems) and (2) risk of all-cause and/or cause-specific mortality. A critical appraisal of the included studies will be undertaken. A synthesis of the findings will include the characteristics of the included studies, critical appraisal and a summary of the key findings in texts and visually in tables. Where appropriate, meta-analyses and subgroup analyses will be performed.

**Ethics and dissemination:**

This study is exempt from ethics approval, as it does not include identifiable human data. The outcomes of the proposed review will be shared in national/international conferences, published in a peer-reviewed journal and disseminated to new and existing networks.

**PROSPERO registration number:**

CRD42023477494.

**STRENGTHS AND LIMITATIONS OF THIS STUDY**

- This systematic review will examine excess mortality in pooled and a range of separate mental disorder categories.
- The study designs will be representative of general populations in geographically defined areas worldwide. In eligible studies, the exposure (mental disorders) will be identified according to structured, semistructured or diagnostic interviews.
- The resulting findings are likely to inform future prevention and control measures.
- Potential limitations include the availability and heterogeneity of the existing evidence and varying methodological quality.

## Introduction

 There is an increasing public health focus on the excess mortality in people with mental disorders.^1^,^3^ Consequently, there are also renewed calls for improved solutions to prevent premature mortality that is associated with mental disorders.^1^,^3^ However, barriers to improve solutions include gaps in existing evidence syntheses.^1^

In their seminal systematic review and meta-analysis, Walker *et al* estimated that eight million deaths per year were attributable to mental disorders involving mood, anxiety and psychoses.^4^ In addition, for people with mood, anxiety and psychotic disorders, the magnitude of risk for non-natural causes of death compared with natural causes was greater, whereas natural causes still accounted for the majority of deaths.^4^ Importantly, over four successive decades, the gap between mortality rates among people with mental disorders and others appeared to increase. A review on a similar topic examined mood disorders, anxiety disorders and schizophrenia in relation to the risk of suicide, specifically in longitudinal studies representative of the general population.^5^ All mental disorders of interest significantly predicted the risk of suicide, with an adjusted risk ratio (RR) of 7.64 (4.3–13.58) for major depressive disorders. A separate review reported that the pooled standardised mortality ratio (SMR) was 3.08 (2.88–3.31) for people with schizophrenia and other non-affective psychotic disorders relative to age-specific and sex-specific rates in the general population (sample sources included patient registers, administrative/claims data and the general population).^6^ However, excess mortality is not understood equally across specific forms of mental ill health in the general population. Thus, new evidence syntheses that address existing gaps are needed.

### Gaps in the literature

We performed a preliminary search in PROSPERO and Medline Complete (see online supplemental tables 1 and 2). We did not identify any review that examined excess mortality in people with different mental disorders such as mood, anxiety, eating, substance use, psychotic and personality disorders in epidemiological studies, representative of the general population. In addition, it remains unclear whether the excess mortality in mental disorders has increased over time. Finally, we did not identify a review that chiefly identified mental disorders using consistent approaches such as diagnostic or semi-structured interviews.

We propose to address this knowledge gap by undertaking a systematic review of the existing literature to examine excess mortality in pooled and a range of mental disorder categories in nationally representative studies or studies that are broadly representative of general populations in geographically defined areas worldwide (eg, in national surveys or community-based studies), which use diagnostic or semistructured interviews.

Separately, the ‘first wave’ of the COVID-19 pandemic might have contributed to a surge of mental health challenges for individuals worldwide. Incipient data have shown that excess mortality in mental disorders appears to have been exacerbated by the pandemic.^7^ In a cohort of 167 122 individuals with mental disorder diagnoses from prospective real-time health records as part of the South London and Maudsley NHS Foundation Trust, mental disorders were associated with significantly increased mortality relative to the general population before and throughout the pandemic period.^7^ While there was an observed peak in mortality in the study population that coincided with a spike in the incidence of COVID-19 (in the UK between March and June 2020), deaths from all non-COVID-19 causes rose in people with specific mental disorders (eg, substance use disorders) compared with the general population.^7^ Thus, synthesising the emerging data on this topic is also needed to inform future prevention and control measures.

### Aims

We propose to systematically review the evidence from nationally representative population-based cohort studies of adults that investigate the following:

1. Mortality rates (all-cause and/or cause-specific) in people with mental disorders (pooled and specific groups of mood, anxiety, eating, substance use, psychotic and personality disorders) compared with people without these mental disorders.
2. Whether excess mortality (all-cause and/or cause-specific) among people with mental disorders has increased over time.
3. Whether the COVID-19 pandemic exacerbated the excess mortality among people with mental disorders.
4. Methodology and social factors that may predict or moderate excess mortality among people with mental disorders.

It is anticipated that the data generated will provide rationale and bolster support for policymakers and healthcare providers to respond to the burden of excess mortality.

## Methods and analyses

### Inclusion and exclusion criteria

This protocol is registered with PROSPERO (CRD42023477494). It was developed according to the Preferred Reporting Items for Systematic review and Meta-Analysis Protocols (PRISMA-P).^8^ Studies will be considered eligible if they investigate mental disorders and mortality in non-experimental designs (eg, case-control or cohort studies) with a follow-up length of ≥1 year. Non-eligible study designs include experimental, qualitative and cross-sectional studies. Specifically, the inclusion and exclusion criteria are shown according to the population, exposure and outcome framework:^9^

#### Population

##### Inclusions

Eligible study samples will be representative of the general population residing in a geographically defined area (eg, national surveys or community-based studies) including participants aged 18 years and over.

##### Exclusions

Participants aged under 18 years and studies of specific population groups—people identified as underserved or vulnerable (eg, veterans, refugees, prisoners, people experiencing homelessness, pregnant women)—will be excluded. Therefore, samples derived from routinely collected administrative data sources (eg, registries, insurance/claims records, providers of services) will be excluded.

### Exposure(s)

#### Inclusions

Studies will be eligible if they examine mental disorders according to structured, semistructured or diagnostic interviews by trained non-clinicians or clinicians (eg, physicians, psychiatrists or psychologists). Two-phase approaches to assessment involving screening, followed by a diagnostic interview, will be eligible. Eligible diagnostic systems include the International Classification of Diseases and Related Conditions and the Diagnostic and Statistical Manual of Mental Disorders.

#### Exclusions

Symptomatology and self-reported mental disorders will be excluded. Types of somatisation/somatic symptom disorders may be considered if they meet the eligibility exposure criteria. However, the types of conditions which are classified as diseases of the musculoskeletal system and connective tissue, or chronic pain will be excluded. Given the population inclusion criteria, mental disorder diagnoses according to registries, insurance/claims records and/or providers of services will be excluded.

### Outcome(s)

The primary outcome is all-cause mortality according to linked mortality databases (eg, national death index/registers). The secondary outcome of interest is cause-specific mortality, which will be dependent on data availability (ie, deaths due to cardiovascular diseases, neoplasms, respiratory diseases and intentional and unintentional injuries). In addition, mortality ascertained from community informants will be eligible.

### Other exclusions

The following exclusions will also be applied:

- Publications that are comments, editorials, viewpoints or abstracts only (published in any language).
- Studies that do not examine mental disorders according to the criteria as defined in the section.
- Studies that do not examine mortality as outlined in the section.

### Information sources

Records of peer-reviewed studies will be searched in Medline Complete, CINAHL Complete, APA PsycInfo via the EbscoHost platform, and Embase. Depending on the available resources and the number of articles retrieved that are published in languages other than English, translations might be undertaken. Sources of grey literature will include published theses/dissertations. Further details are presented in the following section on the search strategy.

### Search strategy

First, we confirmed no existing reviews (ongoing or published) by searching PROSPERO (see online supplemental table 1). We undertook a preliminary scope of peer-reviewed literature in Medline Complete using the EBSCOhost platform on 20 November 2023, yielding 17 457 results (from inception up until November 2023; see online supplemental table 2).

The preliminary search for Medline Complete will be further developed in consultation with a liaison librarian and translated for Embase, CINAHL Complete and PsycInfo databases. The final search strategy will be evaluated using the Peer Review of Electronic Search Strategies checklist. In addition, the reference lists of eligible studies will be searched using Scopus and handsearched if required. The authors of the studies considered eligible may be contacted to make data clarifications/requests.

Grey literature will be searched using ProQuest Dissertations databases. There will be no date restrictions for eligible studies.

The complete search strategy and results will be presented in the review.

### Citation management

The search strategy will be implemented, and records will be managed by one reviewer, with the assistance of a liaison librarian. The records will be exported and uploaded to a reference management software (ie, Covidence) with duplicates removed.^10^

### Selection process

Before screening, the review team will pilot the eligibility and exclusion criteria on a random sample of the records retrieved. Good agreement will be determined if the two reviewers achieve a consensus rate of 75% based on include/exclude decisions and reasons for the exclusion on a sample of the records. If there are discrepancies of 75% or greater, the review team will discuss potential issues and make resulting modifications to the inclusion criteria if necessary.

### Screening

Teams of at least two reviewers will screen the records and undertake full-text review of the articles for eligibility. Potential discrepancies at the screening or full-text stage will be resolved by the same reviewers and/or in consensus with the review team. Next, eligible records will be inspected for their publication date and data source; in the case of potential overlap from studies derived from the same data source, the most recent study or the study with the longest follow-up will be included. The reasons for exclusion will be presented from the full-text review stage.

### Data collection process

#### Critical appraisal of individual studies

At least two reviewers will independently undertake critical appraisal of the included studies using the Joanna Briggs Institute critical appraisal tools; these tools have been selected as they are designed to assess a range of study designs.^11^ Any potential disagreements will be solved by consensus with the review team. Appropriate criteria will be applied to determine the quality and levels of evidence for the reported results.^12^

#### Data extraction

The data extraction plan for this protocol was developed in consultation with a statistician (MM). The planned data items for extraction are presented in table 1. Briefly, at least two reviewers will extract and validate data concerning the source, eligibility criteria, methods, setting, participants/exposures, comparison group, outcomes and mortality estimates. Any potential discrepancies will be resolved by the review team. The data will be entered into an Excel file.

**Table 1:** Data extraction plan

Source	Report ID Study ID Review Author ID Citation details	
Eligibility	Eligibility and exclusion criteria	
Methods	Study design Year of baseline (duration/follow-up time) 1 year ≥1 year and <5 years ≥5 years and <10 years ≥10 years Recruitment Sampling Participation and follow-up rate(s)	
Setting	Representative of general populations in a geographically defined area Nationally Representative study/survey Community-based study/survey	
Population and exposure	Total number (subgroups) Pertinent sociodemographics Age Sex Country Socioeconomic status Ethnicity WHO geographical regions	Identification of mental disorders Mental disorder diagnosis Comorbidity Diagnostic criteria Identification method Diagnostic interviews (type) Rater (non-clinician/clinician)
Comparison group	Control or comparison Age Sex Birth country Socioeconomic status Ethnicity WHO geographical regions Method for determining comparison status	
Outcomes	Primary outcomes: All-cause mortality Cause-specific mortality Natural (ICD codes) Non-natural (ICD codes) Specific categories (ICD codes) May be considered: Total number of deaths/rates Years lived with disability Population attributable risk	**Identification method (mortality**) Linked mortality register Community informant
Estimate of effect/results	Relative risk (RR)/risk ratio (RR) HR OR 95% CIs Other: Standardised mortality ratio Person–time data	

ICDInternational Classification of Diseases

### Data synthesis and analysis

#### Synthesis

The characteristics of the included studies, critical appraisal scores and descriptions of the main findings will be presented in text and visually using tables and figures.

#### Meta-analysis

Where data are available, mental disorder categories of mood, anxiety, substance use, eating, psychotic and personality disorders will be analysed in relation to the primary outcome(s). Hazard ratios (HRs), risk ratios (RRs), standardised mortality ratios (SMRs) or ORs with 95% CIs will be considered the main outcome statistics. The most fully adjusted model(s) from each report will be extracted and reported in tables and/or used in pooled analyses. Where possible, measures of mortality risk will be transformed to enable comparisons (ie, when incident rates are less than 10% and where the value of odds is similar to risk).^13^ The significance level will be set to 0.05. Random-effects meta-analytic models will be performed on the pooled statistics in Stata 17. Meta-regression or comparisons of subgroups will be employed to investigate the effects of the COVID-19 pandemic and time trends in additional analyses to address the secondary aims.

Heterogeneity will be identified and measured from the I^2^ statistic. Further subgroup analyses may also be performed to examine potential sources of heterogeneity including methodological factors (eg, relating to the study design, setting, population and assessment of mental disorders) and individual/social factors (eg, involving age, sex, socioeconomic status).

Publication bias will be examined by visually inspecting funnel plots.

Further information regarding the analyses will be presented in the final review.

### Presenting and reporting results

The proposed review will adhere to the PRISMA and the Meta-analysis of Observational Studies in Epidemiology (MOOSE) guidelines.^14^ A PRISMA flow diagram will present the screening and selection process. The discussion will include a summary of the major findings, limitations of the included studies and review, and potential policy and practice implications.

## Discussion

The proposed systematic review will provide a comprehensive synthesis of the evidence on the topic of mental disorders and excess mortality. Thus, it may contribute to reducing the barriers to create improved solutions to prevent premature mortality in people with mental disorders. Synthesising the existing epidemiological data from broadly representative studies of general populations may inform policymakers and healthcare providers and elicit a response to the burden of excess mortality. For example, it has been highlighted elsewhere that a heightened focus on the management of physical health and suicide prevention and risk reduction is particularly needed in the context of future pandemic response planning and intervention for people with mental ill health.^7^

In terms of possible limitations, there is the potential for inconsistent quality in the methodology and reporting of the studies to be included in the review. For example, the potential for pooled analyses will be dependent on the availability and reporting of appropriate comparable study designs, methods to identify and assess mental disorders and analytical approach. Furthermore, there is the possibility of a ‘healthy’ bias in the identified studies, which might underestimate mortality estimates.

## Ethics and dissemination

This study is exempt from ethics approval or consent procedures, as it does not involve the inclusion of identifiable human data.

The findings will be submitted for publication in a peer-reviewed scientific journal and presented at relevant conferences in the field. Plain language summaries of the key findings will be prepared and disseminated to existing peer and social networks.

### Patient and public involvement

Patient and public involvement was not required for the development of this protocol.

## supplementary material

10.1136/bmjopen-2024-084797 online supplemental file 1
