# Supplementary material for: Mental disorders and excess mortality: a systematic review protocol
Source: BMJ Open. 2025 Mar 15;15(3):e084797. doi: 10.1136/bmjopen-2024-084797 (PMC11911691; doi:10.1136/bmjopen-2024-084797)
Supplement: online supplemental file 1 [file bmjopen-15-3-s001.pdf]

Supplementary Table 1: PROSPERO search terms

mental disorders OR serious mental illness OR severe mental illness OR depression OR anxiety OR personality disorder OR eating disorder OR substance use disorder OR bipolar disorder AND mortality or mortality rate AND Epidemiologic OR Systematic Review OR Meta-Analysis OR Review of reviews

Note: In total, the search yielded 2,149 records. Of the potentially relevant records (n=11), two reviews focused specifically on anxiety disorders, three on eating disorders, four on bipolar disorders and/psychotic disorders, one on personality disorders, and one on mental and behavioral disorders with respect to mortality risk.

Supplementary Table 2: Preliminary search and results of peer-reviewed literature conducted in Medline Complete via EbscoHost

Search line	Terms	Results
**56**	S53 AND S54 AND S55	17,457
**55**	S46 OR S47 OR S48 OR S49 OR S50 OR S51 OR S52	2,537,412
**54**	S43 OR S44 OR S45	1,018,807
**53**	S1 OR S2 OR S3 OR S4 OR S5 OR S6 OR S7 OR S8 OR S9 OR S10 OR S11 OR S12 OR S13 OR S14 OR S15 OR S16 OR S17 OR S18 OR S19 OR S20 OR S21 OR S22 OR S23 OR S24 OR S25 OR S26 OR S27 OR S28 OR S29 OR S30 OR S31 OR S32 OR S33 OR S34 OR S35 OR S36 OR S37 OR S38 OR S39 OR S40 OR S41 OR S42	1,914,635
**52**	AB (case* and control*)	576,596
**51**	TI (case* and control*)	51,812
**50**	(MH "Case-Control Studies+")	1,456,486
**49**	(MH "Epidemiologic Methods")	31,617
**48**	AB (cohort*)	831,523
**47**	TI (cohort*)	175,551
**46**	(MH "Cohort Studies+")	2,537,227
**45**	(MH "Mortality") OR (MH "Mortality, Premature")	50,970
**44**	AB (mortality)	956,877
**43**	TI (mortality)	164,834
**42**	AB (severe or serious) AND ("mental illness*")	12,830
**41**	TI (severe or serious) AND ("mental illness*")	5,493
**40**	AB (“psychotic disorder*")	10,317

**39**	AB (schizophrenia)	105,873
**38**	AB (psychosis)	40,171
**37**	TI (“psychotic disorder*")	2,435
**36**	TI (schizophrenia)	75,436
**35**	TI (psychosis)	27,787
**34**	AB (“personality disorder*”)	21,437
**33**	TI (“personality disorder*”)	11,283
**32**	AB (alcohol or drug or substance) AND (use or abuse or disorder*)	1,027,063
**31**	TI (alcohol or drug or substance) AND (use or abuse or disorder*)	294,794
**30**	AB (orthorexia)	392
**29**	AB (bulimia)	7,476
**28**	AB (anorexia)	30,570
**27**	AB ("eating disorder*")	23,271
**26**	TI (orthorexia)	324
**25**	TI (bulimia)	3,493
**24**	TI (anorexia)	13,122
**23**	TI ("eating disorder*")	12,642
**22**	AB (“adjustment disorder")	1,267
**21**	AB (“posttraumatic stress")	25,168
**20**	AB ("panic disorder")	9,010
**19**	AB (“obsessive compulsive”)	18,668
**18**	AB (phobia)	8,657

**17**	AB (anxiety)	256,298
**16**	TI (“adjustment disorder")	263
**15**	TI (“posttraumatic stress")	11,316
**14**	TI ("panic disorder")	4,705
**13**	TI (“obsessive compulsive”)	10,782
**12**	TI (phobia)	3,494
**11**	TI (anxiety)	70,380
**10**	AB (mania)	9,884
**9**	AB (manic)	10,066
**8**	AB (bipolar)	69,606
**7**	AB (depressive)	143,815
**6**	AB (depression)	398,160
**5**	TI (mania)	4,207
**4**	TI (manic)	3,331
**3**	TI (bipolar)	31,888
**2**	TI (depressive)	45,022
**1**	TI (depression)	130,539

Supplementary Table 2: Preliminary search and results of peer-reviewed literature conducted in Medline Complete via EbscoHost

Search line	Terms	Results

**56**	S53 AND S54 AND S55	17,457
**55**	S46 OR S47 OR S48 OR S49 OR S50 OR S51 OR S52	2,537,412
**54**	S43 OR S44 OR S45	1,018,807
**53**	S1 OR S2 OR S3 OR S4 OR S5 OR S6 OR S7 OR S8 OR S9 OR S10 OR S11 OR S12 OR S13 OR S14 OR S15 OR S16 OR S17 OR S18 OR S19 OR S20 OR S21 OR S22 OR S23 OR S24 OR S25 OR S26 OR S27 OR S28 OR S29 OR S30 OR S31 OR S32 OR S33 OR S34 OR S35 OR S36 OR S37 OR S38 OR S39 OR S40 OR S41 OR S42	1,914,635
**52**	AB (case* and control*)	576,596
**51**	TI (case* and control*)	51,812
**50**	(MH "Case-Control Studies+")	1,456,486
**49**	(MH "Epidemiologic Methods")	31,617
**48**	AB (cohort*)	831,523
**47**	TI (cohort*)	175,551
**46**	(MH "Cohort Studies+")	2,537,227
**45**	(MH "Mortality") OR (MH "Mortality, Premature")	50,970
**44**	AB (mortality)	956,877
**43**	TI (mortality)	164,834
**42**	AB (severe or serious) AND ("mental illness*")	12,830
**41**	TI (severe or serious) AND ("mental illness*")	5,493
**40**	AB (“psychotic disorder*")	10,317
**39**	AB (schizophrenia)	105,873
**38**	AB (psychosis)	40,171
**37**	TI (“psychotic disorder*")	2,435

**36**	TI (schizophrenia)	75,436
**35**	TI (psychosis)	27,787
**34**	AB (“personality disorder*”)	21,437
**33**	TI (“personality disorder*”)	11,283
**32**	AB (alcohol or drug or substance) AND (use or abuse or disorder*)	1,027,063
**31**	TI (alcohol or drug or substance) AND (use or abuse or disorder*)	294,794
**30**	AB (orthorexia)	392
**29**	AB (bulimia)	7,476
**28**	AB (anorexia)	30,570
**27**	AB ("eating disorder*")	23,271
**26**	TI (orthorexia)	324
**25**	TI (bulimia)	3,493
**24**	TI (anorexia)	13,122
**23**	TI ("eating disorder*")	12,642
**22**	AB (“adjustment disorder")	1,267
**21**	AB (“posttraumatic stress")	25,168
**20**	AB ("panic disorder")	9,010
**19**	AB (“obsessive compulsive”)	18,668
**18**	AB (phobia)	8,657
**17**	AB (anxiety)	256,298
**16**	TI (“adjustment disorder")	263

**15**	TI (“posttraumatic stress")	11,316
**14**	TI ("panic disorder")	4,705
**13**	TI (“obsessive compulsive”)	10,782
**12**	TI (phobia)	3,494
**11**	TI (anxiety)	70,380
**10**	AB (mania)	9,884
**9**	AB (manic)	10,066
**8**	AB (bipolar)	69,606
**7**	AB (depressive)	143,815
**6**	AB (depression)	398,160
**5**	TI (mania)	4,207
**4**	TI (manic)	3,331
**3**	TI (bipolar)	31,888
**2**	TI (depressive)	45,022
**1**	TI (depression)	130,539
