# Supplementary material for: Mental disorders and excess mortality: a systematic review protocol
Source: BMJ Open. 2025 Mar 15;15(3):e084797. doi: 10.1136/bmjopen-2024-084797 (PMC11911691; doi:10.1136/bmjopen-2024-084797)
Supplement: Supplementary file 2 [file bmjopen-2024-084797.reviewer_comments.pdf]

PEER REVIEW HISTORY

BMJ Open publishes all reviews undertaken for accepted manuscripts. Reviewers are asked to complete a checklist review form and are provided with free text boxes to elaborate on their assessment. These free text comments are reproduced below.

ARTICLE DETAILS

Title (Provisional)

Mental disorders and excess mortality: A systematic review protocol

Authors

Quirk, Shae; Koivumaa-Honkanen, Heli; Stuart, Amanda L; Mohebbi, Mohammadreza; Honkanen, Risto; Heikkinen, Jeremi; Berk, Michael; Pasco, J; Williams, Lana

---

VERSION 1 - REVIEW

**Reviewer**	**1**
**Name**	**Ali, Suhailah**
**Affiliation**	**The University of Queensland**
**Date**	**23-Feb-2024**
**COI**	**None**

Methods

- You've stated 'Studies will be considered eligible if they are prospective' – does this mean you're excluding retrospective studies? A lot of mortality studies are retrospective e.g. using record linkage to match a pre-existing cohort with national death registers.

- Inclusion/exclusion criteria – you've used the terms 'nationally representative population-based' and 'general population participants', and stated you will be excluding 'participants recruited from specialized settings including mental health' – does this mean you will not be including any inpatient samples, and only using community-based studies? I think you need to be clearer on your study population, and provide justification for your choices. For example, inpatient samples are likely to capture a signification portion of severe mental disorder cases.

- Critical appraisal – have you decided which specific tools/criteria you will be using?

- Data analysis – you've stated 'Hazard ratios (HRs), standardized mortality ratios (SMRs), or odds ratios (ORs) with 95% confidence intervals (CIs) will be considered the main outcome statistics' – will you be treating these as equivalent measures of mortality risk, and if so have

you considered converting the SMRs, HRs and ORs to relative risks where possible, to make them comparable?

- Data analysis – where studies report estimates with several levels of adjustment, which estimates will you include in your analysis?

- Your research aims include 'Which methodology and social factors predict, or moderate excess mortality among people with mental disorders' – in your data analysis section, you have not stated which factors you are going to test.

Discussion

- Limitations – you state 'there is the potential for inconsistent quality in the methodology and reporting of the cohort studies to be included' – please elaborate on this, and what the implications are for your study.

**Reviewer**	**2**
**Name**	**Gaab, Jens**
**Affiliation**	**University of Basel**
**Date**	**19-Mar-2024**
**COI**	**none**

I think this is a needed analysis which would clarify the role and importance of mental disorders for mortality. But even though you include an comprehensive array of mental disorders, you do not include somatoform/medically unexplained symptoms/syndromes (including fatigue, pain, etc). I am fully aware that this neither represents a clearcut category nor are these syndromes spared from controversy, but considering their prevalence and also the dispute regarding their etiology, it would be good to either include them in your analysis or at least to discuss why you do not include them.

VERSION 1 - AUTHOR RESPONSE

Friday, 21 June 2024

Dear Shona Reeves, Executive Editor BMJ Open

RE: bmjopen-2024-084797 - "Mental disorders and excess mortality: a systematic review protocol"

Thank you for the opportunity to revise our manuscript. Please find our responses to the Editor, and Reviewers 1 and 2 within this document. The authors' responses are sign posted in bold and red text—and in tracked changes throughout the revised manuscript.

Thank you for considering our manuscript for publication at BMJ Open.

Shae Quirk on behalf of the authors: Heli Koivumaa-Honkanen, Amanda L Stuart, Mohammadreza Mohebbi, Risto Honkanen, Jeremi Heikkinen, Michael Berk, Julie A. Pasco, and Lana J. Williams.

Reviewer: 1
Dr. Suhailah Ali, The University of Queensland

Comments to the Author:
Methods

1. ***You've stated 'Studies will be considered eligible if they are prospective' – does this mean you're excluding retrospective studies? A lot of mortality studies are retrospective e.g. using record linkage to match a pre-existing cohort with national death registers.***

Author response: Section 2.1 Eligibility criteria and exclusions has been revised. The eligibility criteria in section 2.1.1 have been amended to reflect the various analytic approaches to addressing the research question.

See lines 111-114:

- Studies will be considered eligible if they are non-experimental, representative or population-based, with a follow-up length of ≥1 year. Other than the exposure, the comparison groups will be similar and drawn from the same study population. Non-eligible study designs include experimental, qualitative, and cross-sectional studies.

2. ***Inclusion/exclusion criteria – you've used the terms 'nationally representative population-based' and 'general population participants', and stated you will be excluding 'participants recruited from specialized settings including mental health' – does this mean you will not be including any inpatient samples, and only using community-based studies? I think you need to be clearer on your study population, and provide justification for your choices. For***

example, inpatient samples are likely to capture a signification portion of severe mental disorder cases.

Author response: Commentary in the literature highlights that existing syntheses of the evidence have identified cases of mental disorders in studies of both inpatient and population-based samples—which arguably conflates the population mortality due to mental disorders[1]. We set out to overcome methodological limitations of existing reviews, by a focus on, where practical, nationally representative or population-based studies. To capture more severe mental disorders, a pre-existing population-based cohort that is linked with national psychiatric and death registers will be considered eligible (in line with the reviewer's comment in bullet point one.) In addition, we intend to explore the potential effects of study characteristics on mortality estimates, for example, cases identified by registers versus semi-structured clinical interviews.

[1]Raven M. Analysis of Mortality in Mental Disorders. JAMA Psychiatry. 2015 Nov;72(11):1150. doi: 10.1001/jamapsychiatry.2015.1362. PMID: 26398919.

Text has been amended in the relevant sections:

- Lines 119-120. Studies will be considered if they examine population-based participants aged 18 years and over. Register-based studies that examine entire populations will be considered.
- Lines 122-125. Participants aged under 18 years, and participants recruited based on their status of being admitted to mental health-related care including hospital inpatients, residential facilities, treatment centers, outpatients, or who were recruited from other clinically relevant settings such as community mental health centers, primary or secondary, aged care, and palliative care).
- Lines 130-133. Studies will be eligible if they examine diagnosed mental disorders according to semi-structured clinical interviews, or otherwise identified diagnoses according to a relevant health professional. Diagnoses obtained from registries that are linked to existing/established population-based cohorts will be considered.

3. Critical appraisal – have you decided which specific tools/criteria you will be using?

Author response: Text has been added to address this comment.

See lines 191-193:

- At least two reviewers will independently undertake critical appraisal of included studies using the Joanna Briggs Institute (JBI) critical appraisal tools; these tools have been selected as they are designed to assess a range of study designs[9].

4. ***Data analysis – you've stated 'Hazard ratios (HRs), standardized mortality ratios (SMRs), or odds ratios (ORs) with 95% confidence intervals (CIs) will be considered the main outcome statistics' – will you be treating these as equivalent measures of mortality risk, and if so have you considered converting the SMRs, HRs and ORs to relative risks where possible, to make them comparable?***

Author response: Text has been added in response to this comment.

See lines 211-213:

- Where possible, measures of mortality risk will be transformed to enable comparisons (i.e., when incident rates are less than 10% and where the value of odds are similar to risk) [11].

5. ***Data analysis – where studies report estimates with several levels of adjustment, which estimates will you include in your analysis?***

Author response: Text has been added in response to this comment.

See lines 210-213:

- Hazard ratios (HRs), standardized mortality ratios (SMRs), or odds ratios (ORs) with 95% confidence intervals (CIs) will be considered the main outcome statistics; the most fully adjusted model(s) from each report will be extracted and reported in tables and/or used in pooled analyses.

6. ***Your research aims include 'Which methodology and social factors predict, or moderate excess mortality among people with mental disorders' – in your data analysis section, you have not stated which factors you are going to test.***

Author response: Text has been added in section 2.8.2.

See lines 217-220:

- Heterogeneity will be identified and measured from the I^2 statistic. Subgroup analyses may also be performed, and to examine potential sources of heterogeneity including methodological factors (e.g., related to study design, setting, population, and assessment of mental disorders), and individual/social factors (e.g., involving age, sex, socioeconomic status, and ethnicity).

Discussion

- ***Limitations – you state 'there is the potential for inconsistent quality in the methodology and reporting of the cohort studies to be included' – please elaborate on this, and what the implications are for your study.***

Author response: Text has been added to elaborate on potential limitations.

See lines 238-241:

- For example, the potential for pooled analyses will be dependent upon the availability and reporting of appropriate comparable study designs, methods to identify and assess mental disorders, and analytic approach. Furthermore, there is the possibility of a "healthy" bias in population-based cohort studies, which might underestimate mortality estimates.

Reviewer: 2
Prof. Jens Gaab, University of Basel

Comments to the Author:

- ***I think this is a needed analysis which would clarify the role and importance of mental disorders for mortality. But even though you include an comprehensive array of mental disorders, you do not include somatoform/medically unexplained symptoms/syndromes (including fatigue, pain, etc). I am fully aware that this neither represents a clearcut category nor are these syndromes spared from controversy, but considering their prevalence and also the dispute regarding their etiology, it would be good to either include them in your analysis or at least to discuss why you do not include them.***

Author response: Text has been added in section 2.1.3 to address this comment.

See lines 137-140:

- Types of somatization/somatic symptom disorders may be considered if they meet the eligible exposure criteria. However, types of conditions involving chronic pain such as fibromyalgia, which are classified as diseases of the musculoskeletal system and connective tissue in the ICD will be excluded.

Reviewer: 1
Competing interests of Reviewer: None

Author response: Noted.

Reviewer: 2
Competing interests of Reviewer: none

Author response: Noted.

VERSION 2 - REVIEW

**Reviewer**	**1**
**Name**	**Ali, Suhailah**
**Affiliation**	**The University of Queensland**
**Date**	**16-Jul-2024**
**COI**	**None**

Thank you for addressing all of my comments, I'm happy with all of your responses except for the one around inclusion/exclusion criteria (sorry to be pedantic!).

• I'm not sure I fully understand your justification. You've stated 'existing syntheses of the evidence have identified cases of mental disorders in studies of both inpatient and population-based samples—which arguably conflates the population mortality due to mental disorders". The reference you've provided is critiquing a study that applied mortality estimates derived from clinical samples to population-based estimates; the issues here was not the different sample sources included in the meta-analysis, but rather generalising or applying their mortality risk estimates to the general population (when the authors estimated population attributable risk). This reference therefore does not really support your choice, and I'm not sure what you mean by conflating the population mortality?

• It is, however, still a valid choice, and has been done before e.g. Moitra M, Santomauro D, Degenhardt L, Collins PY, Whiteford H, Vos T, Ferrari A. Estimating the risk of suicide

associated with mental disorders: A systematic review and meta-regression analysis. J Psychiatr Res. 2021 May;137:242-249. doi: 10.1016/j.jpsychires.2021.02.053; and Oakley P, Kisely S, Baxter A, Harris M, Desoe J, Dziouba A, Siskind D. Increased mortality among people with schizophrenia and other non-affective psychotic disorders in the community: A systematic review and meta-analysis. J Psychiatr Res. 2018 Jul;102:245-253. doi: 10.1016/j.jpsychires.2018.04.019. I think you just need a clearer justification, to also be provided in the main text. You should also be more explicit when you say ‘population-based’ e.g. are you referring specifically to studies that are representative of the general population of a given location (e.g. community-based studies)?

• You’ve also said that register-based studies will be considered i.e. cohorts that are linked with national psychiatric registers. These can be based on inpatient and/or outpatient diagnoses (e.g. Nordic registries); therefore, this then conflicts with your updated population exclusion criteria. There are also studies using linked primary and secondary care e.g. in the UK, and nationally representative studies based on insurance or health service databases, which again, conflicts with your exclusion criteria in terms of ‘clinically relevant settings’. You’ve also said under exposure inclusions ‘identified diagnoses according to a relevant health professional’, which again seems conflicting with your population exclusions.

VERSION 2 - AUTHOR RESPONSE

Shauna Heffernan, PhD, Research Editor, BMJ Open
Editor(s)' Comments to Author:

- *Please state the systematic review dates in the Methods section, e.g. the databases were searched from inception to November 2023.*

Author response. Please see additional text in lines 207-208.

- *Please include a statement relating to the ethical approval obtained for your study in the Ethics and Dissemination sub-section of the abstract. If ethical approval was not required, please explain why this is the case.*

Author response. Please see additional text in lines 46-47

- *Please revise points 1 and 2 of the 'Strengths and limitations of this study' section of your manuscript (after the abstract). This section should contain up to five short bullet points, no longer than one sentence each, that relate specifically to the methods. The novelty, aims, results or expected impact of the study should not be summarised here.*

Author response. Please see revised and additional text in lines 54-60

- *Please remove the Conclusion section from your manuscript as this is not a required section for study protocols*

Author response. Lines 300-302 have been removed.

Reviewer: 1
Dr. Suhailah Ali, The University of Queensland
Comments to the Author:

- *Thank you for addressing all of my comments, I'm happy with all of your responses except for the one around inclusion/exclusion criteria (sorry to be pedantic!).*

  I'm not sure I fully understand your justification. You've stated 'existing syntheses of the evidence have identified cases of mental disorders in studies of both inpatient and population-based samples—which arguably conflates the population mortality due to mental disorders". The reference you've provided is critiquing a study that applied mortality estimates derived from clinical samples to population-based estimates; the issues here was not the different sample sources included in the meta-analysis, but rather generalising or applying their mortality risk estimates to the general population (when the authors estimated population attributable risk). This reference therefore does not really support your choice, and I'm not sure what you mean by conflating the population mortality?

  It is, however, still a valid choice, and has been done before e.g. Moitra M, Santomauro D, Degenhardt L, Collins PY, Whiteford H, Vos T, Ferrari A. Estimating the risk of suicide associated with mental disorders: A systematic review and meta-regression analysis. J Psychiatr Res. 2021 May;137:242-249. doi: 10.1016/j.jpsychires.2021.02.053; and Oakley P, Kisely S, Baxter A, Harris M, Desoe J, Dziouba A, Siskind D. Increased mortality among people with schizophrenia and other non-affective psychotic disorders in the community: A systematic review and meta-analysis. J Psychiatr Res. 2018 Jul;102:245-253. doi: 10.1016/j.jpsychires.2018.04.019. I think you just need a clearer justification, to also be provided in the main text. You should also be more explicit when you say 'population-based' e.g. are you referring specifically to studies that are representative of the general population of a given location (e.g. community-based studies)?

  You've also said that register-based studies will be considered i.e. cohorts that are linked with national psychiatric registers. These can be based on inpatient and/or outpatient diagnoses (e.g. Nordic registries); therefore, this then conflicts with your updated population exclusion criteria. There are also studies using linked primary and secondary care e.g. in the UK, and nationally representative studies based on insurance or health service databases, which again, conflicts with your exclusion criteria in terms of 'clinically relevant settings'. You've also said under exposure inclusions 'identified diagnoses according to a relevant health professional', which again seems conflicting with your population exclusions.

Author response.

We thank the reviewer for their careful and considerate approach to reviewing our protocol. The reviewer has adeptly identified inconsistencies to the inclusion and exclusion criteria. We have grappled with this and acknowledge their input into resolving it.

The authors have refined the scope of the review to focus on nationally representative, or studies that are broadly representative of general populations in geographically defined areas worldwide (e.g., in national surveys or community-based studies). Therefore, and given this focus, samples deriving from routinely collected administrative data sources such as registries, insurance/claims records, and providers of services will be excluded. In addition, a novel feature of this review will be a focus on identifying exposures (mental disorders) in the eligible studies using structured, semi-structured, or diagnostic interviews.

Therefore, the authors have clarified the rationale, and the inclusion and exclusion criteria as follows:

- **Introduction, lines 70-84**

  An existing review on a similar topic examined mood disorders, anxiety disorders, and schizophrenia in relation to the risk of suicide specifically in longitudinal studies representative of the general population[5]. All mental disorders of interest significantly predicted risk of suicide, with an adjusted risk ratio (RR) of 7.64 (4.3–13.58) for major depressive disorder. A separate review reported the pooled standardized mortality ratio (SMR) was 3.08 (2.88–3.31) for people with schizophrenia and other non-affective psychotic disorders relative to age- and sex specific rates in the general population (sample sources included patient registers, administrative/claims data, and the general population) [6]. However, it is apparent that excess mortality is not understood equally across specific forms of mental ill health in the general population. Thus, new evidence syntheses that address existing gaps are needed.

- **Introduction, lines 87-97**

  We performed a preliminary search in PROSPERO and Medline Complete (see supplementary Tables 1 and 2). We did not identify any review that examined excess mortality in people with a broad range of mental disorders such as mood, anxiety, eating, substance use, psychotic, and personality disorders in epidemiological studies, representative of the general population. In addition, it remains unclear whether excess mortality in mental disorders has increased over time. Finally, we did not identify a review that chiefly identified mental disorders using consistent approaches such as diagnostic or semi-structured interviews.

  We propose to address this knowledge gap by undertaking a systematic review of the existing literature to examine excess mortality in pooled and a range of mental disorders categories in nationally representative, or studies that are broadly representative of general populations in

geographically defined areas worldwide (e.g., in national surveys or community-based studies), which utilize diagnostic or semi-structured interviews.

- **2.1 Inclusion and exclusion criteria, lines 124-158**

This protocol is registered with PROPSERO (CRD42023477494). It was developed according to the Preferred Reporting Items for Systematic review and Meta-Analysis Protocols (PRISMA-P) [8]. Studies will be considered eligible if they investigate mental disorders and mortality in non-experimental designs (e.g., case-control or cohort studies) with a follow-up length of ≥1 year. Non-eligible study designs include experimental, qualitative, and cross-sectional studies. Specifically, the inclusion and exclusion criteria are shown according to the Population, Exposure, Outcome (PEO) framework [9]:

2.1.1 Population

Inclusions

Eligible study samples will be representative of the general population residing in a geographically defined area (e.g., national surveys or community-based studies) including participants aged 18 years and over.

Exclusions

Participants aged under 18 years, and studies of specific population groups—people identified as underserved or vulnerable (e.g., veterans, refugees, prisoners, people experiencing homelessness, pregnant women)—will be excluded. Therefore, samples derived? from routinely collected administrative data sources (e.g., registries, insurance/claims records, providers of services) will be excluded.

2.1.2 Exposure(s)

Inclusions

Studies will be eligible if they examine mental disorders according to a structured, semi-structured, or diagnostic interviews by trained non-clinicians or clinicians (e.g., physician/psychiatrist/psychologist). Two-phase approaches to assessment involving screening, followed by a diagnostic interview will be eligible. Eligible diagnostic systems include the International Classification of Diseases and Related Conditions (ICD) and the Diagnostic and Statical Manual of Mental disorders (DSM).

Exclusions

Symptomatology and self-reported mental disorders will be excluded. Types of somatization/somatic symptom disorders may be considered if they meet the eligible exposure criteria. However, types of conditions which are classified as diseases of the musculoskeletal system and connective tissue, or chronic pain, will be excluded.

2.1.3 Outcome(s)

The primary outcome is all-cause mortality according to linked mortality databases (e.g., national death index/registers). The secondary outcome of interest is cause-specific

mortality, which will be dependent upon data availability (i.e., deaths due to diseases of the cardiovascular system; neoplasms; diseases of the respiratory system; intentional and unintentional injuries etc.). In addition, mortality ascertained from community informants will be eligible.

VERSION 3 - REVIEW

**Reviewer**	**1**
**Name**	**Ali, Suhailah**
**Affiliation**	**The University of Queensland**
**Date**	**15-Sep-2024**
**COI**	

Thanks for your revisions.
